# Robotic right colectomy for hemorrhagic right colon cancer: a case report and review of the literature of minimally invasive urgent colectomy

**DOI:** 10.1186/1749-7922-9-32

**Published:** 2014-04-26

**Authors:** Emanuele Felli, Francesco Brunetti, Mara Disabato, Chady Salloum, Daniel Azoulay, Nicola de’Angelis

**Affiliations:** 1 Digestive Surgery and Liver Transplant Unit, Henri-Mondor Hospital, Université Paris Est – UPEC, Créteil 94010, France

**Keywords:** Hemorrhagic colon cancer, Robotic surgery, Laparoscopic surgery, Emergency surgery, Minimally invasive surgery, Review

## Abstract

Right colon cancer rarely presents as an emergency, in which bowel occlusion and massive bleeding are the most common clinical presentations. Although there are no definite guidelines, the first line treatment for massive right colon cancer bleeding should ideally stop the bleeding using endoscopy or interventional radiology, subsequently allowing proper tumor staging and planning of a definite treatment strategy. Minimally invasive approaches for right and left colectomy have progressively increased and are widely performed in elective settings, with laparoscopy chosen in the majority of cases. Conversely, in emergent and urgent surgeries, minimally invasive techniques are rarely performed. We report a case of an 86-year-old woman who was successfully treated for massive rectal bleeding in an urgent setting by robotic surgery (*da Vinci Intuitive Surgical System®*). At admission, the patient had severe anemia (Hb 6 g/dL) and hemodynamic stability. A computer tomography scanner with contrast enhancement showed a right colon cancer with active bleeding; no distant metastases were found. A colonoscopy did not show any other bowel lesion, while a constant bleeding from the right pre-stenotic colon mass was temporarily arrested by endoscopic argon coagulation. A robotic right colectomy in urgent setting (within 24 hours from admission) was indicated. A three-armed robot was used with docking in the right side of the patient and a fourth trocar for the assistant surgeon. Because of the patient’s poor nutritional status, a double-barreled ileocolostomy was performed. The post-operative period was uneventful. As the neoplasia was a pT3N0 adenocarcinoma, surveillance was decided after a multidisciplinary meeting, and restoration of the intestinal continuity was performed 3 months later, once good nutritional status was achieved. In addition, we reviewed the current literature on minimally invasive colectomy performed for colon carcinoma in emergent or urgent setting. No study on robotic approach was found. Seven studies evaluating the role of laparoscopic colectomy concluded that this technique is a safe and feasible option associated with lower blood loss and shorter hospital stay. It may require longer operative time, but morbidity and mortality rates appeared comparable to open colectomy. However, the surgeon’s experience and the right selection of candidate patients cannot be understated.

## Introduction

During the past 20 years, a rapid evolution of techniques and technology has occurred for colorectal surgery. Several randomized clinical trials have demonstrated that laparoscopic colectomy for cancer has comparable results in terms of the long-term oncologic outcomes of conventional surgery [1,2]. Moreover, a minimally invasive approach offers several advantages, such as reduced blood loss, decreased postoperative pain, decreased morbidity, earlier bowel transit, and shorter hospital stay [1-4]. Nevertheless, laparoscopic surgery has a longer learning curve compared to traditional surgery [5-7].

In the last decade, minimally invasive colorectal surgery has been implemented by the introduction of the robotic approach that has been increasingly performed with a learning curve relatively short [8]. Right hemicolectomy has been proposed as a training procedure in order to gain clinical experience with the robot [9]. The results of robotic surgery, in terms of oncologic outcome and anastomotic leakage, are presently comparable to laparoscopy, but with longer operating times and greater costs. Nonetheless, in high volume and experienced centers, robotic surgery is indicated for difficult cases where open surgery would most likely be indicated or in cases where laparoscopy would have a high risk of conversion [10].

Right colon cancer rarely presents as an emergency. Usually, the most common symptoms are mild anaemia, weight loss, changes in bowel transit and palpable abdominal mass. Patients are mostly aged, with frequent co-morbidities and sometimes malnutrition. Emergency surgery for symptomatic colon cancer is usually performed with the traditional open technique, as the most common clinical scenarios (perforation, occlusion, massive bleeding) [11] do not allow for proper preparation for minimally invasive techniques. However, minimally invasive emergency colectomy performed by laparoscopy has already been described. Laparoscopy appears to offer several advantages also when performed in emergency setting, although major operative difficulties and longer operative time may represent technical drawbacks [12].

To the best of our knowledge, robotic emergency colectomy has not been previously reported in the literature. We describe the case of a patient with bleeding right colonic carcinoma who was operated by robotic surgery in urgent setting. Additionally, we revised the current literature on the role of minimally invasive surgical procedures performed in emergent or urgent settings in patients with colonic malignancy.

## Case presentation

**Figure 1:**
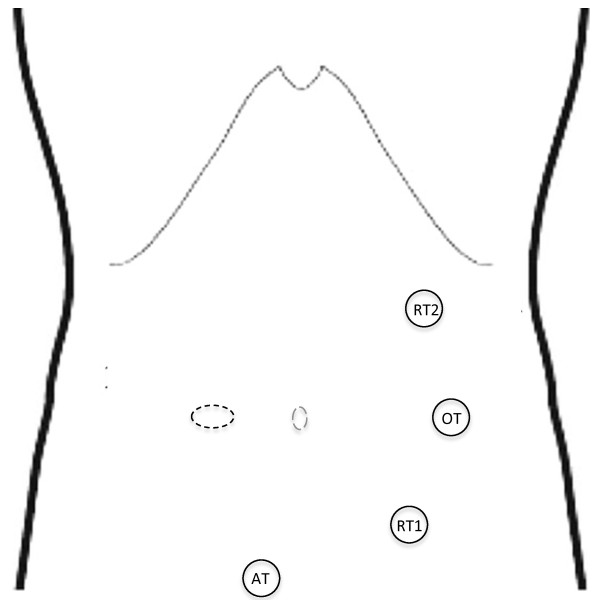
**Schematic representation of the robotic trocar sites.** Precisely one 12-mm optic trocar (OT), two 8-mm robotic working trocars (RT), and one 10-mm assistant trocar (AT). The dotted line represents the double-barreled ileocolostomy.

An 86-year-old woman presented with massive rectal bleeding, severe anemia (Hb 6 g/dL), and hemodynamic stability. The patient had a body mass index of 22 and arterial hypertension. A computed tomography with contrast enhancement showed a right colon carcinoma with active bleeding; no distant metastases were found. The patient was admitted in the intensive care unit (ICU) for resuscitation and blood transfusion, requiring 4 packed red blood cells unit in 24 hours. Laboratory tests showed that PT, creatinine, and urea levels were within the normal ranges. A colonoscopy did not show bowel lesions other than the right colon carcinoma. The constant bleeding from the right colon mass was temporarily arrested by endoscopic argon coagulation. After 12 h surveillance in the ICU, no other bowel bleeding was found and we decided upon an urgent right colectomy without primary anastomosis due to the patient’s poor nutritional status (serum albumin 2.7 g/dL; pre-albumin 112 mg/L) and the important previous body weight loss (>10%), which are recognized risk factors for anastomotic leak and mortality in elderly patients [13-16]. Although the patient was stable, the risk of re-bleeding and related complications was considered high, which led us to decide upon an urgent colectomy. A radical resection was considered achievable with a minimally invasive approach, namely, robotic surgery. The robot present in our department is the *da Vinci Intuitive Surgical System*®. It consists of a vision cart and a surgeon’s console, with the option of a second console for the first assistant surgeon. The patient was placed in a supine position with the legs open. The patient was secured to the operating table with the help of a bean bag, with both arms on the bedside. The robot was on the right side of the patient and the first assistant and the scrub nurse were situated to the patient’s left side. Once the robot is docked, there can be no change to the robot’s or the patient’s position without first undocking the robotic arms. We routinely use only two robotic arms with a third one for the camera (in order to contain surgery-related costs), although three robotic working arms can be used if needed. Robotic trocars were placed on the left mid-clavicular line, and the assistant’s trocar was placed in the hypogastric region below the camera for traction (Figure 1). The first trocar was placed with the Hasson open technique.

**Figure 2:**
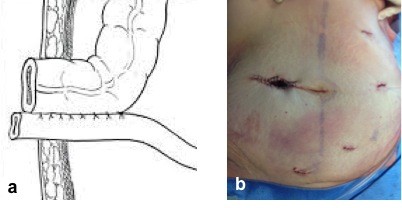
**Double-barreled ileocolostomy. a)** Schematic representation of the double-barreled ileocolostomy; **b)** Picture of the patient’s abdomen showing the incisions and double-barreled ileocolostomy.

The robot was brought from the right side of the patient and docked onto the ports. We routinely use a vessel sealer on the right hand and a bipolar fenestrated grasper on the left robotic arm. The procedure began as any other laparoscopic procedure, with the inspection of the abdominal cavity to evaluate the feasibility of the robotic resection or the presence of other contraindications. The patient was placed in the Trendelenburg position, with a left inclination of 30 degrees. This allowed for good vision of the operating field, exposing the caecum and the terminal part of the ileum, while the small bowel and the omentum were pushed into the upper quadrants. A medial to lateral approach was used. The caecum was grasped and retracted laterally, and the peritoneum was incised in the ileo-caecal fold. The ileo-caecal artery and vein were then dissected and stapled with a vascular stapler. This helped to open the avascular retroperitoneal plane of dissection. The entire right colon was mobilized up to the hepatic flexure. The transverse colon was retracted inferiorly, and the gastrocolic ligament was divided with the help of vessel sealer. The dissection was continued toward the hepatic flexure and the final attachments of the colon to the retroperitoneum were divided. This completed the mobilization of the entire right colon and the robotic part of the procedure. Once completed, the robot was undocked and the site of the double-barreled ileocolostomy was prepared in the right iliac region. The double-barreled ileocolostomy consists in the creation of an ostomy site were both the proximal ileum stump and the transverse colonic stump are tacked together by interrupted 4–0 Vicryl sutures (Figure 2a). The mobilized right colon was entirely exteriorized through the ileocolostomy site (approximately 5 cm) and resected extracorporeally (Figure 2b). No drain was left in the abdomen. The whole procedure took 150 min and the estimated blood loss was 50 ml. The post-operative period was uneventful. The patient was discharged on postoperative day 6 after a re-alimentation and normal bowel transit (achieved at post-operative day 1). The nutritional status improved with specific diet and progressive re-alimentation. The tumor was a moderately differentiated mucinous adenocarcinoma of the colon, classified as pT3N0 (on 17 lymphnodes); no adjuvant chemotherapy was indicated, and surveillance was decided after a multidisciplinary meeting. The ileocolostomy closure was performed three months later with a local approach. Stoma closure was simply achieved by local mobilization at the mucocutaneous junction and extracorporeal anastomosis. At the 5 month follow-up, the patient was well, asymptomatic and without signs of recurrence.

## Review

A literature review of clinical studies focusing on minimally invasive colectomy performed in emergency or urgent setting in adult patients with colon carcinoma was undertaken. For proper identification of studies eligible for the review, the selection criteria were defined before data collection. All types of original studies (randomized and non-randomized controlled clinical trials, case–control studies, cohort studies, case series, case report) that applied laparoscopy, hand-assisted laparoscopy, single-incision laparoscopic surgery (SILS), or robotic surgery for right, transverse, or left colectomy were eligible for inclusion. Only the studies that included at least 1 patient with colon cancer were eligible for inclusion. Clinical trials that applied minimally invasive surgery only for patients with benign diseases were excluded. The primary method to locate potentially eligible studies was a computerized literature search from inception to January 2014 in MEDLINE (through PubMed) and EMBASE databases. In total, 18 articles were identified and retrieved for a more detailed full-text evaluation. Of these, 11 articles were excluded because in their study populations they did not include patients with colon carcinoma. Of the 7 studies included [12,17-22], 2 are comparative studies on patients operated for colon carcinoma only, and the other 5 are case–control studies or case series on samples of patients with both non-malignant and malignant colonic diseases. Data of the included studies are summarized in Table 1. No RCT was found. No study on SILS or robotic surgery for emergency colectomy was found.

**Table 1:** Summary of the studies on minimally invasive colectomy in emergent or urgent settings

**Authors, year**	**Study design**	**Sample size (n)**	**Study population**	**Surgical techniques**	**Conversion rate (LC to OC)**	**Main findings**	**Conclusion of the study**
***Ng et al., 2008*** [19]	Case–control study	43	All patients presented with obstructing right colon carcinoma	The study compared 14 LC vs. 29 OC	Nil (0/14)	LC had longer operative time (187.5 min vs. 145 min), less blood loss, earlier ambulation compared to OC. No group difference was found for time to return of gastrointestinal function, duration of hospital stay (4 days for LC vs. 6 days for OC), and post-operative morbidity (28.6% for LC vs. 55.2% for OC). Overall mortality was nil.	Emergency LC for obstructing right-sided colonic carcinoma is feasible and safe.
***Champagne et al., 2009*** [18]	Case series	20	18 patients were operated for non-malignant diseases and 2 patients for colon carcinoma	All patients were operated by LC	10% (2/20): 1 for diverticulitis, 1 for left sided colon carcinoma	The mean operative time was 162 min and the average length of hospital stay was 8 days. There was 1 reoperation and 3 readmissions within 30 days, with no mortality during the follow-up. Six patients required ICU stays after surgery, and 40% of the patients had one or more postoperative complications.	LC is a feasible option in emergency situations once the surgeon has overcome the learning curve in elective LC procedures.
***Stulberg et al., 2009*** [20]	Case–control study	65	55 patients operated for non-malignant diseases, and 10 for colon carcinoma (3 by OC and 7 by LC).	The study compared 40 LC vs. 25 OC	10% (4/40)	The mean operative time was 180 min for OC and 159 min for LC. LC was associated with lower blood and shorter postoperative stay (8 days for LC vs. 11 days for OC). Perioperative mortality rates were similar between groups (1 for LC vs. 3 for OC).	LC is a feasible option in certain emergency situations.
***Catani et al., 2011*** [17]	Matched case–control study	93	81 patients were operated for non-malignant diseases and 12 patients for colon cancer	The study compared 32 LC vs. 61 OC	5.8% (2/32): 2 cases of perforated diverticulitis	No group difference for mortality (0 for LC and 1 for OC) and the mean operative time (189 min for LC vs. 180 min for OC). LC showed lower post-operative morbidity (0% for LC vs. 14.7% for OC) and shorter hospital stay (6 days for LC vs. 8 days for OC).	With increasing experience, LC would be a feasible and an effective option in emergency settings lowering complication rate and length of hospital stay.
***Ballian et al., 2012*** [22]	Propensity Score-matched case–control study	3552	26.6% of patients in the LC group and 14.4% in the OC group were operated for colon or rectum carcinoma. The remaining for different non-malignant diseases.	The study compared 341 LC vs. 3211 OC	Not reported	LC was associated with longer operative time (142 min vs. 122 min) and shorter hospital stay (11.2 days vs. 15 days) compared to OC. The need for intraoperative blood transfusion, the postoperative morbidity, the 30-day reoperation rates, and the mortality were comparable between groups.	LC with primary anastomosis performed in emergency setting has postoperative morbidity and mortality rates comparable to those seen with OC. LC is associated with longer operative time but reduces the postoperative length of hospital stay.
***Koh et al., 2013*** [12]	Matched case–control study	46	36 patients were operated for non-malignant disease and 10 patients for colon carcinoma (4 by OC and 6 LC)	The study compared 23 LC (15 of which were LHC) vs. 23 OC	17.4% (4/23)	LC was associated with longer operative time (175 min for LC vs. 145 min for OC). The duration of hospitalization (6 days for LC vs. 7 days for OC) and the postoperative morbidity rates were similar between groups. Three patients in each group required postoperative ICU stays or reoperations. Overall mortality was nil. The LC did not incur a higher cost.	Emergency LC in a carefully selected patient group is safe. Although the operative times were longer, the postoperative outcomes were comparable to those of the OC.
***Odermatt et al., 2013*** [21]	Propensity Score-matched case–control study	108	All patients presented with colonic or rectosigmoid junction cancer	The study compared 36 LC vs. 72 OC	8% (3/36) 2 cases of advanced T4 cancers needing extensive resection; 1 case of cancer of transverse colon operated by a general surgeon lacking experience in laparoscopy	LC was associated with a greater number of lymph nodes harvested (17 vs. 13) and a shorter hospital stay (7.5 vs. 11.0 days) compared to OC. The overall 3-year survival rate was 51% in the LC group and 43% in the OC group; the 3-year recurrence-free survival rate was 35% in the LC group and 37% in the OC group, without group difference.	Selective emergency LC for colon cancer performed by experienced specialist colorectal surgeons is not inferior to open surgery with regard to short- and long-term outcomes. LC resulted in a shorter length of hospital stay.

LC stands for laparoscopic colectomy; LHC for laparoscopic hand-assisted colectomy; OC for open colectomy; ICU for intensive care unit.

Overall, the 7 studies evaluating laparoscopic colectomy in emergency or urgent setting concluded that this technique is a safe and feasible option associated with lower blood loss and shorter hospital stay. Laparoscopy may require longer operative time, but morbidity and mortality rates appeared comparable to open colectomy. The conversion rate ranged from 0 to 17%.

Previous studies on the role of a laparoscopic colectomy in treating patients with acute colitis from inflammatory bowel disease or iatrogenic perforation following colonoscopy were able to demonstrate the safety, feasibility and benefits of the laparoscopic approach [23-25]. However, data on the specific case of laparoscopic colectomy for obstructed or hemorrhagic colon carcinoma are rare, and caution should be paid before drawing conclusions because the available studies investigated only small or heterogeneous samples of patients most of the times presenting with a high variety of surgical indications and diagnosis (5/7 studies included patients operated for both malignant and non-malignant pathologies).

Notwithstanding, emergency laparoscopy seems a valuable option but all studies stressed the importance of the surgeon’s experience in elective colorectal laparoscopic procedures and the role of patient selection. It remains under debate which are the precise criteria to select the adequate candidates for minimally invasive colectomy in emergent or urgent settings.

## Conclusions

Right colon cancer may present as an emergency, although this occurs in a minority of patients. A minimally invasive approach can be used if the general conditions of the patient are adequate and the vital prognosis is not affected by a longer procedure or a delayed operation. Robotic surgery still does not have a definite role in colorectal surgery, but its indication is growing constantly. Usually performed for specific sub-groups of elective patients, robotic surgery may also be successfully used in urgent settings with good postoperative and oncologic outcomes.

### Consent

Written informed consent was obtained from the patient for publication of this Case Report and any accompanying images. A copy of the written consent is available for review by the Editor-in-Chief of this journal.

## Abbreviations

ICU: Intensive care unit; LC: Laparoscopic colectomy; LHC: Laparoscopic hand-assisted colectomy; OC: Open colectomy; RTC: Randomized clinical trial.

## Competing interests

All authors have no financial or non-financial competing interest to disclose.

## Authors’ contributions

EF contributed to data acquisition and drafted the manuscript. Nde’A and FB carried out the robotic surgical procedure and were involved in the drafting and critical revision of the manuscript. MD and CS contributed to the data acquisition and manuscript revision. DA revised the manuscript critically and agreed to be accountable for all aspects of the manuscript related to the accuracy or integrity of any part of the work. All authors gave their final approval of this manuscript version to be published.

## Authors’ information

EF: MD, Consultant in General Surgery.

FB: MD, Consultant in Upper and Lower Gastrointestinal Surgery.

CS: MD, Consultant in Hepato-biliary and liver transplantation.

MDS: MD, Resident in General Surgery.

DA: MD, PhD, Head of Digestive Surgery and Liver Transplantation Unit.

Nde’A: MD, PhD(c), Research Fellow in Hepato-biliary and Digestive Surgery.
